# Relationship between fatal road traffic injury rates and Human Development Index in Iran 

**Published:** 2019-08

**Authors:** Alireza Zemestani, Hamid Soori

**Affiliations:** ^*a*^Department of Epidemiology, School of Public Health and Safety, Shahid Beheshti University of Medical Sciences, Tehran, Iran.; ^*b*^Safety Promotion and Injury Prevention Research Center, School of Public Health and Safety, Shahid Beheshti University, Tehran, Iran.

## Abstract

**Background:**

Road Traffic Injuries (RTI) are one of the biggest public health problems in the world and the first cause of lost years of life due to early death in Iran. The aim of this study was evaluating association between fatal road traffic injury rates and Human Development Index in Iran.

**Methods:**

In this study to estimate the mortality rates resulting from fatal RTIs in provinces of Iran; the data recorded on the scene of the accident by Traffic Police of I.R. Iran (RAHVAR Police), in 2014-201 5 have been used. The association between mortality rates of RTIs was evaluated on Human Development Index (HDI) and its components. For determining of distribution of mortality, we used Arc-GIS10 software. Global Moran I Index was used in order to find out about clustering of mortality.

**Results:**

From the point of view of mortality rates based on vehicle registration plate, Alborz province with a death rate of 14.1 and North Khorasan with 4.8 per 100000 person had the highest and lowest levels respectively. The general Moran I index indicates lack of clustering aggregation of deaths caused by RTI (Moran I=-.20, P=.09).

**Conclusions:**

Since more than half of the fatal accidents were related to passenger cars, the government must seriously consider the safety of vehicles as well as road safety and investments to enhance the safety of cars and roads.

**Keywords:**

Road Traffic Injuries, Human Development Index (HDI), Moran’s I

